# Kunkecin A, a New Nisin Variant Bacteriocin Produced by the Fructophilic Lactic Acid Bacterium, *Apilactobacillus kunkeei* FF30-6 Isolated From Honey Bees

**DOI:** 10.3389/fmicb.2020.571903

**Published:** 2020-09-16

**Authors:** Takeshi Zendo, Chihiro Ohashi, Shintaro Maeno, Xingguo Piao, Seppo Salminen, Kenji Sonomoto, Akihito Endo

**Affiliations:** ^1^Department of Bioscience and Biotechnology, Faculty of Agriculture, Graduate School, Kyushu University, Fukuoka, Japan; ^2^Department of Food, Aroma and Cosmetic Chemistry, Faculty of Bioindustry, Tokyo University of Agriculture, Hokkaido, Japan; ^3^Functional Foods Forum, University of Turku, Turku, Finland

**Keywords:** bacteriocins, lantibiotics, nisin, *Apilactobacillus kunkeei*, *Melissococcus plutonius*, fructophilic lactic acid bacteria

## Abstract

*Apilactobacillus kunkeei* FF30-6 isolated from healthy honey bees synthesizes the bacteriocin, which exhibits antimicrobial activity against *Melissococcus plutonius*. The bacteriocin, kunkecin A, was purified through three-step chromatography, and mass spectrometry revealed that its relative molecular mass was 4218.3. Edman degradation of purified kunkecin A showed only the N-terminal residue, isoleucine. Hence, alkaline alkylation made the subsequent amino acid residues accessible to Edman degradation, and 30 cycles were sequenced with 11 unidentified residues. Whole genome sequencing of *A. kunkeei* FF30-6, followed by Sanger sequencing, revealed that the genes encoding the proteins involved in lantibiotic biosynthesis were within the plasmid, pKUNFF30-6. Most of the identified proteins exhibited significant sequence similarities to the biosynthetic proteins of nisin A and its variants, such as subtilin. However, the kunkecin A gene cluster lacked the genes corresponding to *nisI*, *nisR*, and *nisK* of the nisin A biosynthetic gene cluster. A comparison of the gene products of *kukA* and *nisA* (kunkecin A and nisin A structural genes, respectively) suggested that they had similar post-translational modifications. Furthermore, the structure of kunkecin A was proposed based on a comparison of the observed and calculated relative molecular masses of kunkecin A. The structural analysis revealed that kunkecin A and nisin A had a similar mono-sulfide linkage pattern. Purified kunkecin A exhibited a narrow antibacterial spectrum, but high antibacterial activity against *M. plutonius*. Kunkecin A is the first bacteriocin to be characterized in fructophilic lactic acid bacteria and is the first nisin-type lantibiotic found in the family *Lactobacillaceae*.

## Introduction

Fructophilic lactic acid bacteria (FLAB) are only found in fructose-rich niches, such as flowers and fruits, and *Fructobacillus* spp. and *Apilactobacillus kunkeei*, formerly classified as *Lactobacillus kunkeei* (Zheng et al., 2020), are representatives of this group (Endo and Okada, 2008; Endo et al., 2012). Recent studies revealed that the genotypic and phenotypic characteristics of FLAB enable them to adapt to the fructose-rich niches (Endo et al., 2009, 2015, 2018). *A. kunkeei* was originally isolated from wine (Edwards et al., 1998) and was recently characterized as one of the major components of the gut microbiota in honey bee queens and larvae (Endo and Salminen, 2013; Vojvodic et al., 2013; Anderson et al., 2018). The species has been linked to the nectar and hive materials of honey bees (Anderson et al., 2013; Kwong and Moran, 2016). As *A. kunkeei* has a symbiotic relationship with the host insects, it has potential probiotic and paratransgenic applications to honey bees (Rangberg et al., 2015; Arredondo et al., 2018). A previous study reported that the culture supernatant from an *A. kunkeei* isolate exhibited anti-*Melissococcus plutonius* activity (Endo and Salminen, 2013), the causative agent of European foulbrood in honey bee larvae (Arai et al., 2012). Furthermore, this antibacterial activity was inhibited by a treatment with proteases (Endo and Salminen, 2013), which suggested the proteinaceous nature of this substance.

Bacteriocins are ribosomally synthesized antimicrobial peptides that exhibit bactericidal or bacteriostatic activity (Cotter et al., 2005b; Alvarez-Sieiro et al., 2016). Various bacterial species, including lactic acid bacteria (LAB), produce bacteriocins, whereas properties for bacteriocin production vary according to strains. The application of bacteriocins, particularly those derived from LAB, in food safety has been attracting increasing interest because most of them are active against several food spoilage bacteria and foodborne pathogens and are also easily degraded by gut proteases (Cleveland et al., 2001; Perez et al., 2014). Moreover, LAB bacteriocins have been evaluated as an alternative antibiotic agent for clinical applications (Heunis et al., 2013; Perez et al., 2014).

Nisin is the most-studied LAB bacteriocin. It is a class I bacteriocin that is also referred to as lantibiotics and is widely used as a safe food preservative (Delves-Broughton et al., 1996). Several natural variants of nisin are produced by strains belonging to *Lactococcus lactis*, such as nisin A (Gross and Morell, 1971), nisin Z (Mulders et al., 1991), nisin Q (Zendo et al., 2003; Fukao et al., 2008), and nisin F (de Kwaadsteniet et al., 2008), in addition to subtilin produced by strains of *Bacillus subtilis* (Banerjee and Hansen, 1988). Natural nisin variants produced by strains of other genera have recently been reported, such as nisin U (Wirawan et al., 2006), nisin H (O’Connor et al., 2015), nisin O (Hatziioanou et al., 2017), nisin J (O’Sullivan et al., 2020), and nisin P (Garcia-Gutierrez et al., 2020) by *Blautia* spp., *Staphylococcus* spp., and *Streptococcus* spp. All nisin variants share a basal structure consisting of five mono-sulfide bridges (lanthionine rings), and three dehydrated amino acid residues, which result from post-translational modifications but have some amino acid substitutions. The biosynthesis of nisin A requires eleven genes in three transcription units (Kuipers et al., 1995; de Ruyter et al., 1996). After the synthesis of NisA, the precursor of nisin A, NisB catalyzes the dehydration of Ser and Thr residues in NisA to dehydroalanine (Dha) and dehydrobutyrine (Dhb), respectively. NisC then catalyzes the cyclization of the dehydrated residues with five Cys residues to form lanthionine (Lan) and 3-methyllanthionine (MeLan), respectively. Modified NisA containing five mono-sulfide bridges and three dehydrated residues is secreted from the producer cell through NisT [an ATP-binding cassette (ABC) transporter]. The 23-amino-acid-long leader sequence of NisA is then cleaved by NisP (protease) outside the cell, and the 34-amino acid mature nisin A is released. The two-component regulatory system, NisK (histidine kinase)/NisR (response regulator) upregulates transcription units to enhance the synthesis of nisin A. Mature nisin A can also serve as an autoinducer. NisI (membrane protein) and NisFEG (ABC transporter) are two independent self-immunity systems that protect producer cells against the antimicrobial activity of nisin A. The other nisin variants are synthesized by a similar mechanism employing similar biosynthetic proteins (Yoneyama et al., 2008).

The honey bee isolate *A. kunkeei* FF30-6 produces an antibacterial peptide that exhibits anti-*M. plutonius* activity (Endo and Salminen, 2013). In the present study, we purified and characterized the structure and activity of a novel bacteriocin, which we named kunkecin A. The gene cluster encoding the proteins involved in bacteriocin biosynthesis was identified through a whole genome analysis of *A. kunkeei* FF30-6. The results of our analysis revealed that kunkecin A is a variant of nisin A, which had not yet been reported in the family *Lactobacillaceae.* Kunkecin A had a narrow antimicrobial spectrum but exhibited high antimicrobial activity against a few bacteria originating from honey bees, including *M. plutonius*.

## Results

### Purification and Mass Determination of the Bacteriocin

**FIGURE 1:**
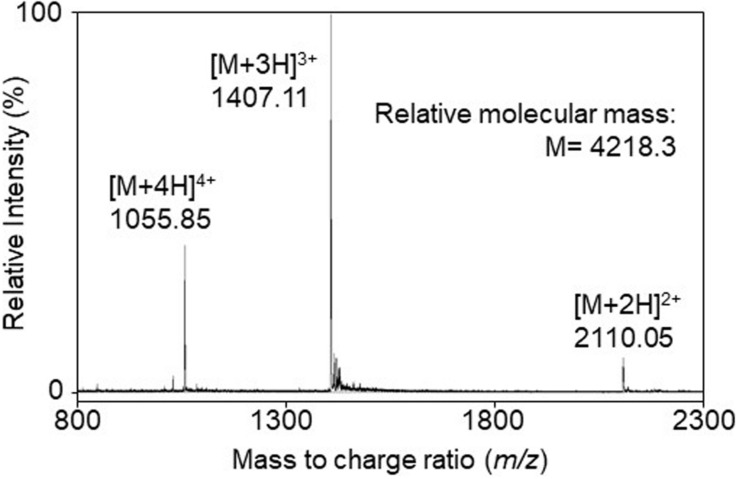
The electrospray ionization time-of-flight (ESI-TOF) mass spectrum of the purified bacteriocin, kunkecin A, produced by *A. kunkeei* FF30-6. Multiple charged molecular ions were detected and are indicated.

The bacteriocin produced by *A. kunkeei* FF30-6 was purified from the culture supernatant using a three-step chromatography procedure. The active fraction containing the purified bacteriocin was obtained in the final step of reverse-phase high-performance liquid chromatography (HPLC). The active fraction was used to characterize the structure and assess its antibacterial activity. Electrospray ionization time-of-flight mass spectrometry (ESI-TOF MS) revealed that the relative molecular mass of the purified bacteriocin was 4218.3 (Figure 1). It was considered to be a novel bacteriocin and termed kunkecin A, because, to the best of our knowledge, its relative molecular mass has not been previously reported for any other bacteriocins in the literature or databases dedicated to bacteriocins such as Bactibase (Hammami et al., 2010) and BAGEL4 (Van Heel et al., 2018).

### Amino Acid Sequence Analysis of Kunkecin A

Edman degradation of purified kunkecin A detected only the N-terminal residue, isoleucine, but no other residues. This result suggested that there was a modified residue at the second position, such as a dehydrated amino acid residue, which inhibited Edman degradation. The purified peptide was further treated with alkaline 2-mercaptoethanol to enable the degradation reaction to access the modified residues in kunkecin A, following the methods described by Meyer et al. (1994). After 30 cycles of Edman degradation of the treated peptide, we obtained the following amino acid sequence: IXXYVLXXPG XIXGRLMGXN NKXKXXHXHS, where X indicates the cycle at which no amino acid was identified. This result strongly suggested that kunkecin A is a lantibiotic and that the second residue and subsequent X residues were post-translationally modified.

### Identification of Kunkecin A Biosynthetic Genes in the Whole Genomic Sequence of *A. kunkeei* FF30-6

**FIGURE 2:**
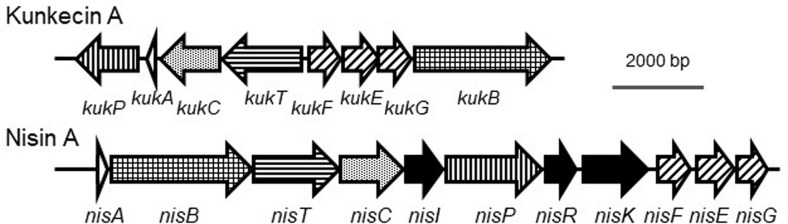
Comparison of biosynthetic gene clusters of kunkecin A and nisin A. The putative kunkecin A biosynthetic genes are labeled with the same pattern as each corresponding gene in the nisin A biosynthetic cluster. No genes corresponding to *nisI*, *nisR*, or *nisK* (filled with black) were found in the kunkecin biosynthetic gene cluster.

A previous study reported the draft genome sequence of *A. kunkeei* FF30-6 (NZ_BDDX00000000) with 25 contigs (Maeno et al., 2016). One of the contigs (contig no. 12) was suspected to be a putative plasmid sequence because it had a gene-encoding DNA replication initiator protein A. In the present study, we used Sanger sequencing to determine the possible plasmid structure. We obtained a circular plasmid, termed pKUNFF30-6, which consisted of 19,498 bp (Supplementary Figure S1 and Supplementary Table S1). The complete plasmid sequence was analyzed using the DDBJ Fast Annotation and Submission Tool (DFAST,^1^) (Tanizawa et al., 2016), which revealed that eight bacteriocin-related genes were located on the plasmid (accession number, AP019008). The amino acid sequences of the gene products exhibited significant similarities to those of the proteins involved in the biosynthesis of nisin A (Table 1). This putative gene cluster consisting of eight consecutive genes (*orf10-17* of pKUNFF30-6) lacked the genes corresponding to *nisI*, *nisR*, and *nisK* in the nisin A biosynthetic cluster. Half of the genes in the cluster (*orf10-13*) were coded in the opposite direction to the other half, *orf14-17*. This is in contrast to nisin A biosynthetic genes, which were all coded in the same direction (Figure 2). Each gene in the kunkecin A biosynthetic gene cluster was named after the corresponding gene in the nisin A biosynthetic gene cluster (Table 1 and Figure 2).

**TABLE 1:** Putative kunkecin A biosynthetic proteins encoded on genes identified in the plasmid, pKUNFF30-6, harbored by *A. kunkeei* FF30-6.

ORF No.*	Product name	Length (a.a.)	Putative function	Corresponding nisin A biosynthetic protein [identity (%)]
10	KukP	454	Leader peptidase	NisP (31%)
11	KukA	64	Kunkecin A precursor	NisA (54%)
12	KukC	437	Lantibiotic cyclase	NisC (25%)
13	KukT	577	Transporter	NisT (37%)
14	KukF	227	Self-immunity	NisF (48%)
15	KukE	241	Self-immunity	NisE (20%)
16	KukG	245	Self-immunity	NisG (22%)
17	KukB	996	Lantibiotic dehydratase	NisB (24%)

*Gene numbers in the plasmid, pKUNFF30-6 (Supplementary Figure S1 and Supplementary Table S1).

### Proposed Primary Structure of Kunkecin A

**FIGURE 3:**
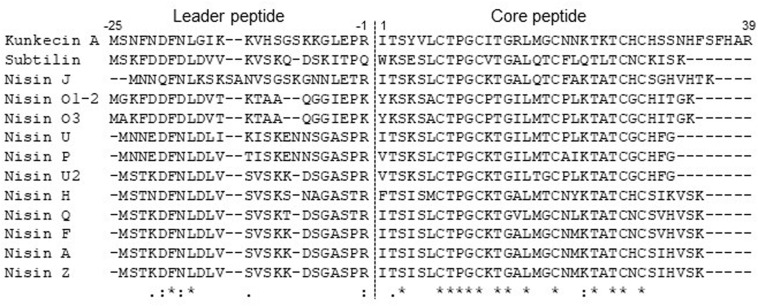
Amino acid sequence alignment of precursor peptides of kunkecin A and nisin variants. Each precursor was aligned using the Clustal Omega program. The dotted line indicates the cleavage site of the leader peptides. Asterisks indicate fully conserved positions. Colons and periods indicate positions conserved by amino acid residues with strong and weak similarity, respectively.

**FIGURE 4:**
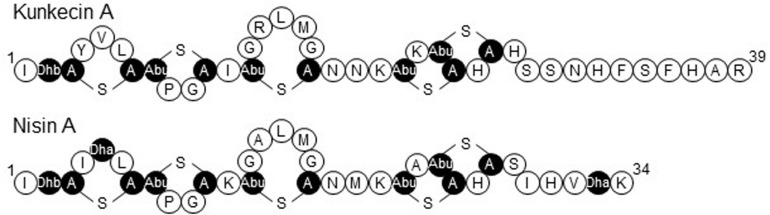
The proposed primary structure of kunkecin A and the primary structure of nisin A. Unusual amino acids generated by post-translational modifications are indicated in black. Dha and Dhb indicate the dehydrated amino acids, dehydroalanine, and dehydrobutyrine, respectively. A-S-A and Abu-S-A indicate lanthionine and 3-methyllanthionine, respectively.

The precursor peptide of kunkecin A, KukA was similar to those of nisin A (NisA) and its natural variants (Figure 3). The cleavage site of the leader peptide of KukA was identified based on the alignment with NisA and the result of Edman degradation, as shown in Figure 3. The difference in the calculated (4327.0) and observed (4218.3) relative molecular masses of the KukA core peptide (Figure 1) indicated that six dehydrations occurred in the core peptide. Additionally, a comparative analysis of the structures of nisin A and kunkecin A revealed that modified residues were located at the position of X in the N-terminal sequence of kunkecin A obtained by Edman degradation. The positions of the modified residues were similar in nisin A, except for the 5th and 33rd residues, at which valine and histidine, respectively, were detected in kunkecin A both by Edman degradation and DNA sequence analysis. These modifications involved six dehydrations and concurred with the observed difference between the calculated and observed relative molecular masses of kunkecin A. Consequently, we proposed the structure of kunkecin A as shown in Figure 4. The structure of kunkecin A includes a dehydrobutyrine, lanthionine, and four 3-methyllanthionine residues with a similar ring pattern to that of nisin A. However, the C-terminal of kunkecin A was longer than that of nisin A.

### Antimicrobial Activity Spectrum of Kunkecin A

The antimicrobial activity of purified kunkecin A was evaluated against a panel of bacterial indicator strains as the minimum concentrations, causing clear inhibition zones by the spot-on-lawn assay. We also compared the antibacterial activity of purified kunkecin A with that of nisin A (Table 2). The antibacterial activity of kunkecin A was two-fold higher than that of nisin A against *M. plutonius*. Honey bee commensals show different sensitivities to the bacteriocins at the species level. *Lactobacillus apis*, *L. kullabergensis*, *Bombilactobacillus mellis*, and *Bifidobacterium asteroides* exhibited markedly higher tolerance (>four-fold) to kunkecin A than to nisin A, whereas others, including *Apilactobacillus apinorum*, *Bombilactobacillus mellifer*, and *Lactobacillus melliventiris*, showed the opposite reactions. The kunkecin A-producing strain, *A. kunkeei* FF30-6, and the nisin A-producer strain, *L. lactis* subsp. *lactis* NCDO 497, were highly tolerant to kunkecin A and nisin A. The tolerance to the kunkecin A and nisin A treatments may be attributed to self-immunity against their own and homologous bacteriocins. The American foulbrood-causing species, *Paenibacillus larvae* PL-1, was tolerant to kunkecin A or nisin A. The minimum concentrations for the inhibition of kunkecin A against some of the indicator strains, including a Gram-negative strain, *Escherichia coli*, were higher than the concentration at which self-immunity was observed.

**TABLE 2:** Antimicrobial spectra of kunkecin A and nisin A.

Indicator strains*	Minimum concentrations for inhibition (nM)	
	Kunkecin A	Nisin A
*Bacillus subtilis* JCM 1465^T^	49	9
*Kocuria rhizophila* NBRC 12708	197	146
*Enterococcus faecalis* JCM 5803^T^	1,578	73
*Enterococcus faecium* TUA 1344L	789	36
*Listeria innocua* ATCC 33090^T^	197	18
*Lapidilactobacillus dextrinicus* JCM 5887^T^	49	9
*Latilactobacillus sakei* subsp. *sakei* JCM 1157^T^	25	9
*Lactiplantibacillus plantarum* subsp. *plantarum* JCM 1149^T^	395	73
*Leuconostoc mesenteroides* subsp. *mesenteroides* JCM 6124^T^	99	9
*Lactococcus lactis* subsp. *lactis* ATCC 19435^T^	197	36
*Lactococcus lactis* subsp. *lactis* NCDO 497 (nisin A-producing LAB)	25,250	582
*Escherichia coli* JM109	12,625	291
**Honey bee commensals**		
*Apilactobacillus kunkeei* JCM 16173	197	146
*Apilactobacillus kunkeei* FMO-1	395	582
*Apilactobacillus kunkeei* FMO-15	197	582
*Apilactobacillus apinorum* JCM 30765^T^	8	582
*Lactobacillus helsingborgensis* JCM 30766^T^	197	291
*Lactobacillus kimbladii* JCM 30767^T^	49	73
*Lactobacillus melliventris* JCM 30770^T^	13	146
*Lactobacillus apis* SH3-5	789	146
*Lactobacillus kullabergensis* SH3-7	98	9
*Bombilactobacillus mellifer* JCM 30768^T^	25	73
*Bombilactobacillus mellis* JCM 30769^T^	789	73
*Bifidobacterium asteroides* SH3-1	49	9
*Bifidobacterium coryneforme* SH6-2	395	291
*Bifidobacterium indicum* SH4-4	3,156	1,164
*Fructobacillus fructosus* FMO-85	395	291
*Apilactobacillus kunkeei* FF30-6 (kunkecin A-producing LAB)	789	582
**Honey bee pathogens**		
*Melissococcus plutonius* ATCC 35311^T^	13	25
*Paenibacillus larvae* PL-1	395	582

*The indicator strains were obtained from the following culture collections: JCM, Japan Collection of Microorganisms, Wako, Japan; NBRC, NITE Biological Resource Center, Chiba, Japan; ATCC, American Type Culture Collection, Manassas, VA; TUA, Tokyo University of Agriculture, Tokyo, Japan; NCDO, National Collection of Dairy Organisms, Reading, United Kingdom. JM109 was purchased from Takara Bio. The other indicator strains were from laboratory collections.

The self-immunity was further examined by the deferred antagonism assay. Against *A. kunkeei* FF30-6, a very tiny inhibition zone with a clear edge was caused by *L. lactis* subsp. *lactis* NCDO 497, but no inhibition zone was formed by itself. On the other hand, against *L. lactis* subsp. *lactis* NCDO 497, no inhibition zones were formed by the both strains.

## Discussion

We previously reported that a culture supernatant of one of the *A. kunkeei* isolates, strain FF30-6, originated from honey bees inhibited the growth of the type strain of *M. plutonius* (Endo and Salminen, 2013). Since *A. kunkeei* is a promising candidate for probiotic and paratransgenic in honey bees, it would be of interest to study its antagonistic activities in honey bee commensals.

The relative molecular mass of kunkecin A is unique among known bacteriocins and within the reported range for LAB bacteriocins and larger than those of most lantibiotics, including nisin A (Cotter et al., 2005a). However, further structural analyses on purified kunkecin A suggested that it shares amino acid sequences and positions of modified residues with nisin A and its variants. The conserved motif sequence (FNLD) of the leader peptide in nisin-group lantibiotics (Plat et al., 2011) was also detected in the kunkecin A leader peptide, except for the final 4th residue, which changed to glycine (Figure 3). The amino acid sequence of the N-terminal in kunkecin A begins with isoleucine and has a dehydrobutyrine at the second position, which is consistent with the results of Edman degradation. The comparative analysis of the observed and calculated relative molecular masses of kunkecin A revealed that the peptide underwent six dehydrations, including dehydrobutyrine at the second position. Furthermore, the comparison of kunkecin A and nisin A strongly suggested that kunkecin A contained totally five mono-sulfide linkages (one lanthionine and four 3-methyllanthionine), which are also found in nisin A. The amino acid sequences of the putative modification enzymes, KukB and KukC, also exhibited high similarities to those of the respective lantibiotic modification enzymes of nisin A and its variants. This result strongly indicated that the ring pattern of kunkecin A was similar to that of the nisin A-group lantibiotics.

In the putative kunkecin A biosynthetic gene cluster, eight genes on the plasmid pKUNFF30-6 encoded putative lantibiotic biosynthetic proteins and the kunkecin A precursor peptide. Putative kunkecin A biosynthetic proteins also exhibited significant sequence similarities to those involved in the biosynthesis of nisin A (Table 1). The function of these proteins was predicted to be similar to that of nisin A biosynthetic proteins. The kunkecin A precursor peptide, KukA, is dehydrated and cyclized by the lantibiotic modification enzymes, KukB and KukC, respectively. Modified KukA is secreted by the ABC transporter, KukT, and the leader peptide of KukA is cleaved by the cell-wall-anchored leader peptidase, KukP, outside the producer cell. The producer cell is protected by the self-immunity proteins, KukF, KukE, and KukG, which comprise another ABC transporter. In contrast to the nisin A biosynthetic gene cluster, the kunkecin A gene cluster lacks the genes corresponding to *nisI*, *nisR*, and *nisK*. NisI is responsible for self-immunity against nisin A (Kuipers et al., 1993), and NisR and NisK form a two-component regulatory system with nisin A as the autoinducer to regulate the biosynthesis of nisin A (Kuipers et al., 1995; de Ruyter et al., 1996). In the reported nisin-variant gene clusters, that of nisin H lacks the gene corresponding to *nisI* (O’Connor et al., 2015), while that of nisin J lacks the genes to *nisI*, *nisR*, and *nisK* (O’Sullivan et al., 2020).

NisI is a membrane-anchored lipoprotein that functions coordinately with the ABC transporter comprising NisF, NisE, and NisG for self-immunity in the producer cell (Stein et al., 2003). NisFEG or SpaFEG, the corresponding system to subtilin, alone is known to be sufficient to impart self-immunity against each cognate lantibiotic (Kuipers et al., 1993; Stein et al., 2003). This finding suggests that the KukFEG system was sufficient to protect producer cells against kunkecin A without the need for a protein corresponding to NisI or SpaI in nisin A or subtilin, respectively. The producer strain, *A. kunkeei* FF30-6 exhibited tolerance to both kunkecin A and nisin A (Table 2), while it was inhibited very weakly by nisin A-producing *L. lactis* NCDO 497 in the deferred antagonism assay. The less cross-immunity of *A. kunkeei* FF30-6 against the nisin A producer can be attributed to high production of nisin A and/or the lack of the NisI homologous protein. Although NisI and SpaI contributes less to total immunity than NisFEG and SpaFEG, respectively, previous studies reported that they protected producer cells from the pore formation activity of their respective cognate bacteriocins (Stein et al., 2005; AlKhatib et al., 2014). Additionally, SpaI has a very similar structure to NisI (Hacker et al., 2015) and interacts with the C-terminal of subtilin (Geiger et al., 2019). The lack of the NisI homologous protein in kunkecin A biosynthesis may be related to the C-terminal extension of kunkecin A and its resulting activity.

During nisin A biosynthesis, nisin A can activate the promoters located upstream of *nisA* and *nisF via* the two-component NisR/NisK regulatory system and trigger the synthesis of nisin A (Kuipers et al., 1995; de Ruyter et al., 1996). No regulatory genes were identified in the kunkecin A biosynthetic gene cluster. Additionally, gene organization in the kunkecin A gene cluster differed from that in the nisin A gene cluster. These two lines of evidence suggest that the regulation of kunkecin A synthesis differs from that of nisin A synthesis.

Kunkecin A exhibited higher antibacterial activity against *M. plutonius* than nisin A, even though these two lantibiotics share structural similarity. However, although they shared a mono-sulfide bridge pattern, kunkecin A lacked two dehydrated residues at positions five and 33 and possessed five extra amino acid residues in the C-terminal. These changes may be responsible for the differences observed in their antibacterial activities.

*Melissococcus plutonius* was one of the most sensitive strains to kunkecin A among the honey-bee-related microbes tested. Antibiotics generally kill pathogens and commensals, which may result in diarrhea and the development of drug-resistant bacterial strains in animals. The long-term antibiotic treatment of honey bee colonies in apiaries has led to the selection of genes that confer antibiotic-resistance to the gut microbiota of the honey bee (Tian et al., 2012). Consequently, the incidence of antibiotic-resistant foulbrood pathogens is high in countries, which use oxytetracycline in apiaries (Murray and Aronstein, 2006; Alippi et al., 2007; Murray et al., 2007; Tian et al., 2012). Furthermore, antibiotic treatments increase the mortality of honey bees (Raymann et al., 2017). The administration of a broad spectrum bacteriocin, such as nisin A, may also affect the gut microbiota in honey bees. The microbiota is crucial for the healthy development of honey bees (Zheng et al., 2017; Raymann et al., 2018). The population of commensal bacteria, such as *Bifidobacterium* is inversely associated with the population of *M. plutonius* in healthy honey bees. Further studies are needed to clarify whether kunkecin A is a useful tool for controlling pathogens in apiaries.

In the present study, we described a novel bacteriocin and lantibiotic, kunkecin A. This is the first bacteriocin reported from FLAB and is the first nisin-type lantibiotic found in the family *Lactobacillaceae*. A more detailed understanding of the regulatory mechanisms underlying kunkecin A biosynthesis may enhance the production of this novel lantibiotic for future applications.

## Materials and Methods

### Strains and Culture Media

The bacteriocin-producing strain, *A. kunkeei* FF30-6 isolated from healthy honey bees (*Apis mellifera mellifera*) (Endo and Salminen, 2013) was cultured in fMRS medium, Lactobacilli MRS broth (MRS; BD Difco, Sparks, MD) supplemented with 2% (w/v) D-fructose (Nacalai Tesque, Kyoto, Japan). *Lapidilactobacillus dextrinicus* JCM 5887^T^ and *M. plutonius* ATCC 35311^T^, used as general indicator strains for bacteriocin activity, were cultured in MRS medium at 30°C and KSBHI medium at 37°C, respectively. KSBHI medium was composed of Brain Heart Infusion (BHI) medium (Oxoid, Hampshire, United Kingdom) supplemented with 20.4 g/L of KH_2_PO_4_ and 10 g/L of soluble starch (Arai et al., 2012). The other bacterial strains that were used as indicator strains for the bacteriocin assay (Table 2) were cultured for 18 h under the optimal conditions recommended by the respective culture collections. All bacterial cultures were stored at –80°C with 15% glycerol and were propagated in the respective media at the recommended temperatures for 18 h before use.

### Antibacterial Activity Assay

The antibacterial activity of bacteriocin was evaluated using the spot-on-lawn method (Ennahar et al., 2001), in which 10 μL of the bacteriocin preparation was spotted onto the bacterial lawn. Regarding general indicator strains, Lactobacilli Agar AOAC (BD Difco) inoculated with an overnight culture of the indicator strain at a density of 10^7^ CFU/mL was overlaid on an MRS agar plate [MRS supplemented with 1.2% (w/v) agar]. KSBHI medium and BHI medium supplemented with 5% (v/v) horse blood and 1.5% (w/v) agar were used for *M. plutonius* and *P. larvae*, respectively, instead of the double layer of Lactobacilli Agar AOAC and MRS agar. After an overnight incubation at the respective temperatures recommended for the indicator strain, the bacterial lawn was checked for inhibition zones. Regarding purified bacteriocins, two-fold serial dilutions of the solution containing a fixed concentration of a bacteriocin were assayed, and the minimum concentrations that resulted in a clear zone of inhibition on each indicator lawn were recorded as the intensity of the antibacterial activity. Each assay was performed in triplicate to confirm reproducibility.

To study a self-immunity against the own bacteriocins, the deferred-antagonism assay was further conducted by using the bacteriocin producers, *A. kunkeei* FF30-6 and *L. lactis* subsp. *lactis* NCDO 497 as indicator strains. The bacteriocin producing strains were stabbed into fMRS agar plate [fMRS supplemented with 1.5% (w/v) agar] and grown at 30°C for 8 h. Then, fMRS agar inoculated with an indicator strain was overlaid on the agar plate. After an overnight incubation, the bacterial lawn was examined for inhibition zones.

### Purification of the Bacteriocin

The bacteriocin was purified using a three-step chromatography procedure that was previously described with minor modifications (Masuda et al., 2011). Purification was performed using four 250-mL cultures (total of 1 L) of *A. kunkeei* FF30-6 grown to the early stationary phase in fMRS medium at 30°C for 8 h with reciprocal shaking at 140 strokes/min. Cells were pelleted by centrifugation at 8000 *g* at 4°C for 20 min. Further, 20 g of activated Amberlite XAD-16 resin (Sigma-Aldrich, St. Louis, MO, United States) was added to the culture supernatant. The resin matrix was shaken slowly at 4°C for 4 h, and was then washed with 200 mL Milli-Q water, followed by 400 mL 50% (v/v) ethanol. The active fraction was eluted with 200 mL 70% (v/v) isopropanol containing 0.1% trifluoroacetic acid. The eluted active fraction was evaporated to 60 mL to remove isopropanol. The sample was then diluted with an equal volume of 50 mM sodium citrate buffer (pH 3.0, CB), and applied to an SP Sepharose Fast Flow cation-exchange chromatography column (internal diameter, 10 mm; length, 100 mm; GE Healthcare, Uppsala, Sweden) equilibrated with 50 mL CB. The column was washed with 50 mL CB and 100 mL of 0.3 M NaCl in CB. The active bacteriocin fraction was eluted with 40 mL of 0.6 M NaCl in CB. This active fraction was applied to a Capcell-Pak C18 MGII S5 column (internal diameter 4.6 mm; length 150 mm; Shiseido, Tokyo, Japan) in an LC-2000Plus HPLC system (JASCO, Tokyo, Japan). The active fractions were eluted at a flow rate of 1 mL/min with a linear gradient of 15–45% (v/v) of acetonitrile in the Milli-Q water-acetonitrile mobile phase containing 0.1% trifluoroacetic acid for 30 min. The main active fraction was further purified using reverse-phase HPLC under the same conditions. Purified active fractions were stored at –30°C. The antibacterial activities of the fractions obtained at each purification step were determined as described above using *L. dextrinicus* JCM 5887^T^ as an indicator strain. Nisin A was purified from a commercial nisin A preparation (Sigma) by using cation-exchange chromatography and reverse-phase HPLC, as described previously (Fujita et al., 2007). Regarding structural characterization, the purified fractions were concentrated using a SpeedVac concentrator (Savant, Farmingdale, NY, United States). To assess antibacterial activity, the solvent was completely removed by lyophilization, and the purified bacteriocin was dissolved in 10% (v/v) dimethyl sulfoxide (DMSO). Peptide concentrations were determined using a Pierce^®^ BCA^TM^ Protein Assay Kit (Takara Bio, Otsu, Japan).

### Mass Spectrometry and Amino Acid Sequencing

The relative molecular masses of the purified fractions were analyzed by ESI-TOF MS using a JMS-T100LC mass spectrometer (JEOL, Tokyo, Japan). Amino acid sequences were determined based on Edman degradation using a PPSQ-31 protein sequencer (Shimadzu, Kyoto, Japan). In further analyses of the N-terminal sequences of peptides containing dehydrated residues and lanthionine, the purified peptide was treated with alkaline 2-mercaptoethanol, following the methods described by Meyer et al. (1994) and subjected to Edman degradation.

### Identification of Bacteriocin-Related Genes

The draft genome sequence of *A. kunkeei* FF30-6 containing 25 contigs was obtained in the previous study with the accession number NZ_BDDX00000000 (Maeno et al., 2016). One of the contigs (contig no. 12), which exhibited plasmid-like genetic characteristics, was further sequenced using the following primers: FF306-c12-F (5′-AAAAGAATAGACAACCACCCA-3′) and FF306-c12-R (5′-CCTTTCTAAGAGGAATATGG-3′). The sequence of the plasmid termed pKUNFF30-6 was analyzed using DFAST^2^ to detect bacteriocin-related genes. Potential genes were further analyzed using the BLAST program of the National Center for Biotechnology Information database^3^. The DNA and amino acid sequences obtained were analyzed using GENETYX-WIN software (GENETYX, Tokyo, Japan). The amino acid sequence alignment was analyzed using Clustal Omega^4^.

## Data Availability Statement

The datasets presented in this study can be found in online repositories. The names of the repository/repositories and accession number(s) can be found below: https://www.ddbj.nig.ac.jp/, AP019008.

## Author Contributions

TZ and AE designed the project, analyzed the data, and wrote the manuscript. CO, SM, and XP performed the experiments and analyzed the data. SS and KS supervised the project. All authors contributed to the article and approved the submitted version.

## Conflict of Interest

The authors declare that the research was conducted in the absence of any commercial or financial relationships that could be construed as a potential conflict of interest.

## Footnotes

1 https://dfast.nig.ac.jp/

2 https://dfast.nig.ac.jp/

3 http://www.ncbi.nlm.nih.gov/BLAST/

4 https://www.ebi.ac.uk/Tools/msa/clustalo/
