# Supplementary material for: Kunkecin A, a New Nisin Variant Bacteriocin Produced by the Fructophilic Lactic Acid Bacterium, Apilactobacillus kunkeei FF30-6 Isolated From Honey Bees
Source: Front Microbiol. 2020 Sep 16;11:571903. doi: 10.3389/fmicb.2020.571903 (PMC7525160; doi:10.3389/fmicb.2020.571903)
Supplement: Supplementary file 1 [file Image_1.pdf]

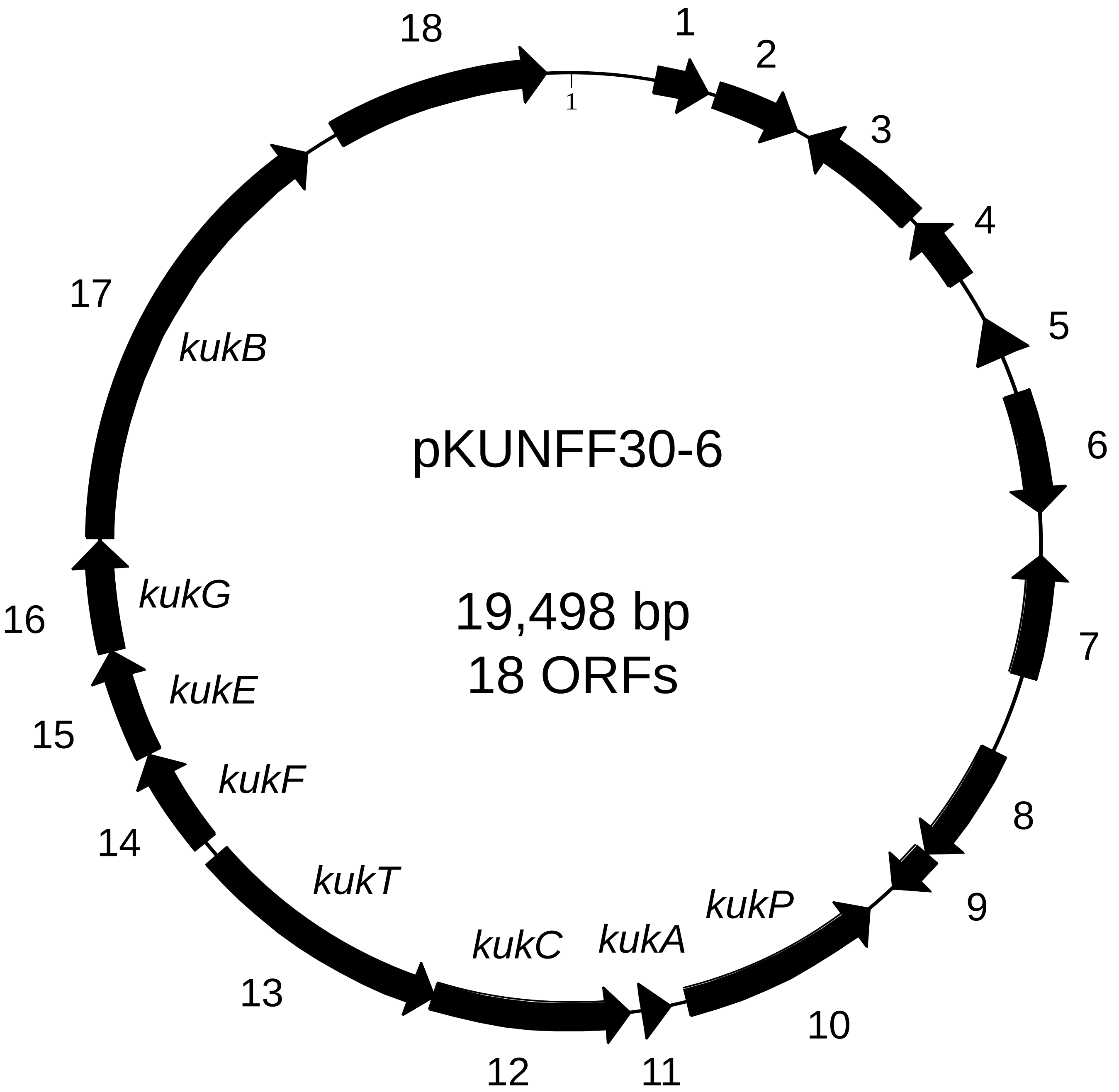

Supplementary Figure S1. Genetic map of plasmid pKUNFF30-6. Putative ORFs are shown as arrows, and the ORF numbers and gene names are indicated outside and inside, respectively.
