# Supplementary material for: Kunkecin A, a New Nisin Variant Bacteriocin Produced by the Fructophilic Lactic Acid Bacterium, Apilactobacillus kunkeei FF30-6 Isolated From Honey Bees
Source: Front Microbiol. 2020 Sep 16;11:571903. doi: 10.3389/fmicb.2020.571903 (PMC7525160; doi:10.3389/fmicb.2020.571903)
Supplement: Supplementary file 2 [file Table_1.pdf]

Supplementary Table S1. ORFs on pKUNFF30-6 and their putative products.

ORF	Location	Gene name	Annotation/Product name	Size (aa)
1	566..928		Hypothetical protein	120
2	979..1557		DNA invertase-like protein	192
3	Complement (1642..2508)		Hypothetical protein	288
4	Complement (2560..3033)		Hypothetical protein	157
5	Complement (3328..3606)		Hypothetical protein	92
6	3867..4667		Hypothetical protein	266
7	Complement (4949..5752)		Hypothetical protein	267
8	6284..7096		Replication initiator protein A	270
9	7093..7413		Hypothetical protein	106
10	Complement (7612..8976)	*kukP*	Leader peptidase, KukP	454
11	Complement (9083..9277)	*kukA*	Lantibiotic kunkecin A precursor, KukA	64
12	Complement (9356..10669)	*kukC*	Lantibiotic cyclase, KukC	437
13	Complement (10662..12395)	*kukT*	ABC transporter protein, KukT	577
14	12511..13194	*kukF*	Self-immunity protein, KukF	227
15	13197..13922	*kukE*	Self-immunity protein, KukE	241
16	13919..14656	*kukG*	Self-immunity protein, KukG	245
17	14667..17657	*kukB*	Lantibiotic dehydratase, KukB	996
18	17885..19342		Hypothetical protein	485
